# 

**Published:** 1858-08

**Authors:** 


### CONTENTS.

ORIGINAL.

Physiological Observations on the Teeth, by Prof. St. John,........................ 59

Extracting Teeth by Galvanism, by Jerome 65

Vulcanite Base, by J. T. T.,............... 69

Metallic Casts, by C. P. Baird,............ 70

Inflammation, by C. C. Dills,.............. 71

PROCEEDINGS OF SOCIETIES.

American Dental Convention,............ 79

Western Dental Society,.................... 88

Crystal Gold Foil, Adhesive Foil, Crystal Gold,.............................. 92

NOTICES AND CUTTINGS.

From..................................94 to 100

Editor's Table,.............................. 100

POETRY.

Without Teeth,.............................. 102

COMMERCIAL.

Currency Bankable in Cincinnati,......... 103

Markets,...................................... 104

Advertisements,............................... 105

